# Histone deacetylase as emerging pharmacological therapeutic target for neuropathic pain: From epigenetic to selective drugs

**DOI:** 10.1111/cns.14745

**Published:** 2024-05-07

**Authors:** Wencui Zhang, Bo Jiao, Shangchen Yu, Caixia Zhang, Kaiwen Zhang, Baowen Liu, Xianwei Zhang

**Affiliations:** ^1^ Department of Anesthesiology and Pain Medicine, Hubei Key Laboratory of Geriatric Anesthesia and Perioperative Brain Health, and Wuhan Clinical Research Center for Geriatric Anesthesia Tongji Hospital, Tongji Medical College, Huazhong University of Science and Technology Wuhan China

**Keywords:** histone deacetylase, histone deacetylase inhibitor, neuroinflammation, neuronal excitability, neuropathic pain

## Abstract

**Background:**

Neuropathic pain remains a formidable challenge for modern medicine. The first‐line pharmacological therapies exhibit limited efficacy and unfavorable side effect profiles, highlighting an unmet need for effective therapeutic medications. The past decades have witnessed an explosion in efforts to translate epigenetic concepts into pain therapy and shed light on epigenetics as a promising avenue for pain research. Recently, the aberrant activity of histone deacetylase (HDAC) has emerged as a key mechanism contributing to the development and maintenance of neuropathic pain.

**Aims:**

In this review, we highlight the distinctive role of specific HDAC subtypes in a cell‐specific manner in pain nociception, and outline the recent experimental evidence supporting the therapeutic potential of HDACi in neuropathic pain.

**Methods:**

We have summarized studies of HDAC in neuropathic pain in Pubmed.

**Results:**

HDACs, widely distributed in the neuronal and non‐neuronal cells of the dorsal root ganglion and spinal cord, regulate gene expression by deacetylation of histone or non‐histone proteins and involving in increased neuronal excitability and neuroinflammation, thus promoting peripheral and central sensitization. Importantly, pharmacological manipulation of aberrant acetylation using HDAC‐targeted inhibitors (HDACi) has shown promising pain‐relieving properties in various preclinical models of neuropathic pain. Yet, many of which exhibit low‐specificity that may induce off‐target toxicities, underscoring the necessity for the development of isoform‐selective HDACi in pain management.

**Conclusions:**

Abnormally elevated HDACs promote neuronal excitability and neuroinflammation by epigenetically modulating pivotal gene expression in neuronal and immune cells, contributing to peripheral and central sensitization in the progression of neuropathic pain, and HDACi showed significant efficacy and great potential for alleviating neuropathic pain.

## Graphical abstract

Histone acetylation modification is governed by the intricate interplay of histone deacetylases (HDACs) and histone acetyltransferases (HATs), where elevated HDAC activity in both neurons and immune cells regulates the expression of genes implicated in pain pathways, promoting neuronal excitability and neuroinflammation, and ultimately, enabling neuropathic pain.

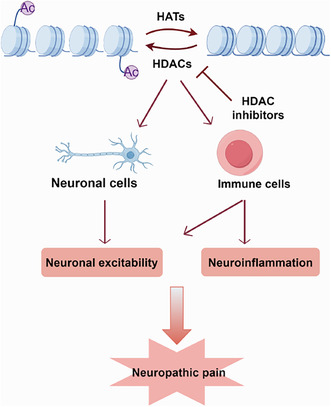

## 1 INTRODUCTION

Neuropathic pain is a chronic and debilitating condition caused by a lesion or disease of the somatosensory nervous system in the periphery or centrally, impacting approximately 7%–10% of the adult population.^1^ Clinically, neuropathic pain typically manifests as aberrant hypersensitivity to noxious stimuli (hyperalgesia), nociceptive responses to non‐noxious stimuli (allodynia) and spontaneous pain without stimuli.^2^ It persists long after the initial injury is resolved, tending to be more refractory than other forms of chronic pain.^3^ Conventional analgesic drugs for neuropathic pain are solely symptomatic and effective in less than half of patients, accompanied by dose‐limiting side effects that influence patients' compliance and hamper the analgesic effects of painkillers.^4^ However, there exists a dearth of comprehensive knowledge regarding the intricate pathogenesis underlying the progression of neuropathic pain and discouraging treatment. Thus, elucidating the exact mechanisms underpinning neuropathic pain and providing novel and therapeutic strategies toward safe and highly efficacious treatment are still urgently needed.

The role of epigenetic adaptations in regulating nociceptive sensitization in the pathogenesis of pain has gained substantial recognition.^5^, ^6^, ^7^ Epigenetic regulation encompasses DNA methylation, histone modifications and noncoding RNAs, potently exerting stable or heritable modification in gene function that persists for a long period, even the next generation and without alteration in the DNA sequence.^8^ Specifically, histone post‐translational modifications (PTM) are among the best‐characterized epigenetic mechanisms, including acetylation, methylation, phosphorylation and ubiquitination. Histone tails protrude from the nucleosome and contain positively charged amino acids, such as lysine and arginine, that undergo consequence of transcriptional repression or activation depending on the residue modified.^9^ Of which, histone acetylation and deacetylation, governed by histone acetyltransferases (HATs) and histone deacetylases (HDACs), are the most extensively characterized posttranslational covalent modifications. By definition, HATs, deemed as “writers”, add acetyl groups to lysine residues on N‐terminal histone tails, whereas HDACs, termed as “erasers”, remove acetyl groups from lysine residues of histone, establishing a tight controlled equilibrium in the dynamic control of acetylation state to archive a specific pattern of gene expression transcription.^10^, ^11^ Mechanistically, HATs reduces the electrostatic affinity between histones and negatively charged DNA, inducing chromatin relaxation to promote gene expression. In contrast, HDACs trigger chromatin condensation and repress gene transcription.^12^, ^13^

Mounting evidence links dysregulated HDAC activity to various diseases, including neurodegenerative diseases, cardiovascular diseases, cancer, as well as neuropathic pain.^5^, ^14^, ^15^, ^16^ Over the past few decades, HDACs have been identified to regulate a plethora of gene transcription involved in neuronal plasticity and neuroinflammation in the peripheral and central nervous system, playing a pivotal role in the onset and maintenance of neuropathic pain.^17^, ^18^, ^19^ Moreover, restoration of these diverse molecular and cellular modifications of HDACs has been shown to reverse a number of aberrant pain‐related genes in the pain epigenome and alleviate neuropathic pain.^20^, ^21^ Nevertheless, a well‐defined causal and mechanistic framework elucidating the precise role of HDACs in the development of neuropathic pain is still lacking. In this review, we outline an overview of the HDAC family, shedding light on their aberrant activity in the context of neuropathic pain, and provide new insights into the dysregulated HDACs in different cells during pain processing. Additionally, we discuss the therapeutic potential of pharmacological HDAC inhibitors in neuropathic pain, as supported by preclinical studies.

## 2 ABERRANT ALTERATIONS OF HDAC FAMILY IN NEUROPATHIC PAIN

So far, HDACs are classified into four categories based on their functions and structures. Class I includes HDAC1, HDAC2, HDAC3 and HDAC8. Class II includes HDAC4, HDAC5, HDAC6, HDAC7, HDAC9 and HDAC10, which can be further classified into IIa (4, 5, 7, 9, and) and IIb (6 and 10). Class III are NAD^+^ dependent HDAC, also termed as sirtuins and includes SIRT1‐SIRT7. Class IV includes HDAC11. HDACs are typically exclusively expressed in the nucleus, except for Class II, which has distinctive characteristics with nucleocytoplasmic shuttling. Various HDACs exhibit distinct and sometimes contradictory functions, and are present in different cell types within the nervous system. A growing sherd of evidence has demonstrated aberrant expression of HDAC subtypes in the dorsal root ganglion (DRG) and spinal cord of various animal models of neuropathic pain (Table 1).

**TABLE 1:** The expression, distribution and interacting partner of HDACs in neuropathic pain.

Class	Type	Model	Expression	Subcellular localization	Interacting partner	References
I	HDAC1	CCI‐induced neuropathic pain rats	DRG ⬆	Glial cells and small neurons	NA	24
		SCI‐induced neuropathic pain mice	DRG ⬆	NA	NA	26
		SNL‐induced neuropathic pain mice	Spinal cord ⬆	Neurons, astrocytes and microglia	RGS10	31
		SNL‐induced neuropathic pain mice	Spinal cord ⬆	Neurons	SOX10	28
	HDAC2	CCI‐induced neuropathic pain rats	DRG ⬆	Large neurons	Kv1.2	24
		CCI‐induced neuropathic pain rats	Spinal cord ⬆	Neurons	GAD65	22
		CCI‐induced neuropathic pain rats	Spinal cord ⬆	Microglia	miR‐183	23
		SNI‐induced neuropathic pain rats	Spinal cord ⬆	Neurons and astrocytes	NA	25
		Paclitaxel‐induced neuropathic pain rats	Spinal cord ⬆	Neurons and astrocytes	YY1	32
	HDAC3	SNL‐induced neuropathic pain rats	Spinal cord ⬆	Neurons and microglia	STAT1	30
		SCI‐induced neuropathic pain rats	Spinal cord ⬆	Neurons and microglia	STAT1, NF‐kB	97
II	HDAC4	CCI‐induced neuropathic pain rats	DRG ⬆	NA	NA	34
		SCI‐induced neuropathic pain mice	DRG ⬆	NA	NEDD4	26
		SNL‐induced neuropathic pain rats	Spinal cord	Neurons	HMGB1	36
	HDAC5	pSNL‐induced neuropathic pain mice	Spinal cord ⬆	Neurons, astrocytes and microglia	SOX10	37
		pSNL‐induced neuropathic pain mice	Spinal cord ⬆	NA	GAD65	71
III	SIRT1	CCI‐induced neuropathic pain rats	Spinal cord ⬇	NA	NA	40
		Streptozotocin‐induced diabetic neuropathic pain rats	Spinal cord ⬇	Neurons	mGluR1, mGluR5	41
	SIRT3	Streptozotocin‐induced diabetic neuropathic pain rats	Spinal cord ⬇	Neurons	FoxO3a	42
		SNI‐induced neuropathic pain mice	Spinal cord ⬇	Neurons	CypD	80
IV	HDAC11	NA	NA	NA	NA	NA

Abbreviations: CCI, chronic constriction injury; CypD, cyclophilin D; DRG, dorsal root ganglion; FoxO3a, transcription factors of the forkhead box class O 3a; GAD65, glutamic acid decarboxylase; HMGB1, high‐mobility group box 1 protein; Kv1.2, the voltage‐gated potassium channel 1.2; mGluR1, metabotropic glutamate receptor 1; mGluR5, metabotropic glutamate receptor 5; NEDD4, neuronal precursor cell‐expressed developmentally down‐regulated 4; NF‐kB, nuclear factor‐kappa B; pSNL, partial sciatic nerve ligation; RGS10, regulator of G protein signaling; SCI, spinal cord injury; SNI, spared nerve injury; SNL, spinal nerve ligation; SOX10, SRY‐box‐containing gene 10; STAT1, the signal transducers and activators of transcription 1; YY1, YY1 transcription factor.

### 2.1 Class I HADCs

In a rat model of neuropathic pain induced by chronic constriction of the sciatic nerve (CCI), the expression of HDAC1 in DRG and HDAC2 in DRG and spinal cord were significant increased.^22^, ^23^, ^24^ In the DRG, HDAC1 was mainly distributed in glial cells and small neurons, seldom colocalizing with NF200‐positive large, myelinated neurons. Conversely, HDAC2 was predominantly present in NF200‐positive neurons.^24^ In the spinal cord, the increased HDAC2 was primarily located in neurons and microglia cells of the spinal dorsal horn.^22^, ^23^ Maiaru et al. demonstrated that spinal HDAC2 was widely expressed in neurons and, to a lesser extent, in astrocytes in naive rats. Intriguingly, the expression of HDAC2 in spinal astrocytes significantly increased following spared nerve injury (SNI)‐induced neuropathic pain, while no difference was observed in neurons.^25^ Moreover, in a mouse model of central neuropathic pain induced by spinal cord injury (SCI), Wang et al. found a significant increase in the expression of HDAC1 in DRG.^26^ Nevertheless, its specific cellular distribution within the DRG remains unclear. In the spinal nerve ligation (SNL)‐induced neuropathic pain model, a series of experiments showed an increase in HDAC1 expression on the ipsilateral of the spinal dorsal horn.^27^, ^28^, ^29^, ^30^ Besides, the increased HDAC1 was expressed in neurons, astrocytes and microglia of spinal cord.^28^, ^31^ It has been observed that the expression of HDAC3 was elevated in both microglia and neurons in the spinal cord following SNL.^30^ Furthermore, in paclitaxel‐induced neuropathic pain rats, Wang et al. showed an enhanced expression of HDAC2 mainly in the spinal neurons, but also sparsely in the astrocytes.^32^

### 2.2 Class II HADCs

Unlike other HDACs that primarily reside in the nucleus, HDAC4 shuttles between the nucleus and cytoplasm.^33^ The increased level of HDAC4 has been observed in the DRG of CCI rats.^34^ However, the total HDAC4 level remained unchanged in the spinal cord of SNL rats.^35^, ^36^ Strikingly, there was a significant increase in cytoplasmic accumulation of HDAC4, which was closely associated with allodynia behavior. Specifically, HDAC4 was primarily located in neurons of the spinal cord. Moreover, the level of HDAC5 was found to be remarkably increased in the DRG of SCI mice.^26^ In a mouse model of partial sciatic nerve ligation (pSNL)‐induced neuropathic pain, Gu et al. observed an elevation of HDAC5 in neurons, microglia, and astrocytes of the spinal dorsal horn.^37^

### 2.3 Class III HADCs

The role of NAD^+^‐dependent HDAC sirtuins has also been investigated.^38^, ^39^ For example, Yin et al. first observed the decreased expression of SIRT1 in the spinal cord of CCI rats.^40^ Then, in high‐fat‐fed/low‐dose streptozotocin‐induced diabetic neuropathic pain (DNP) rats, Zhou et al. showed that the expression of SIRT1 and SIRT3 were considerably decreased and mostly localized in neurons of the spinal cord.^41^, ^42^

### 2.4 Class IV HDACs

HDAC11 is the most recently identified isoform and shares properties with Class I and Class II, which have not been systematically studied in neuropathic pain.

Taken together, these findings suggest that HDACs display differential and dynamic subcellular localization in different neuropathic conditions, suggesting the cell‐specific nature of gene expression epigenetically regulated by HDACs.

## 3 THE CELLULAR AND MOLECULAR MECHANISM OF HDACs IN NEUROPATHIC PAIN

It is widely recognized that nociceptors in the peripheral nervous system (PNS) and neurons in the central nervous system (CNS) are sensitized to various stimuli, also referred to as peripheral sensitization and central sensitization, accompanied by functional and structural changes (plasticity) during the development of neuropathic pain.^43^ Meanwhile, a substantial body of evidence underscores the significant contributions of various types of immune cells, such as glial cells, macrophages and T lymphocytes, to both peripheral and central sensitization.^44^, ^45^ In response to noxious stimulus, these cells can release proinflammatory cytokines, including tumor necrosis factor‐α (TNF‐α), interleukin‐1β (IL‐1β), and IL‐6. Recent compelling evidence suggests that HDACs control the expression of numerous genes in neuronal and non‐neuronal cells, contributing to the nociceptive sensitization and chronification of neuropathic pain.

### 3.1 DRG neurons

Overwhelming evidence supports the involvement of HDAC‐related machineries in the epigenetic regulation of pain‐related ion channels within DRG neurons, including voltage‐gated sodium channels, potassium channels and calcium channels.^17^, ^46^, ^47^ The voltage‐gated sodium channels expressed in DRG, especially Nav1.7 and Nav1.8, are crucial for the hyperexcitability of DRG neurons and nociceptive responses.^48^, ^49^ Neuronal precursor cell‐expressed developmentally down‐regulated 4 (NEDD4), a member of the E3 ubiquitin ligase, has been found to decrease Nav1.7 expression via ubiquitination in chronic post‐surgical pain.^48^ A recent study conducted in SCI‐induced neuropathic pain mice revealed that treatment with an HDAC inhibitor suppressed the expression of HDAC5 and Nav1.7 in DRG neurons, while increasing NEDD4 expression.^26^ They demonstrated that knockdown of HDAC5 upregulated NEDD4 levels by promoting the enrichment of H3K27Ac in the NEDD4 promoter, which subsequently augmented the ubiquitination of Nav1.7 and led to a reduction in Nav1.7 expression in DRG.^26^ On the contrary, Matsushita et al. demonstrated that sustained downregulation of Nav1.8 in DRG neurons in pSNL‐induced neuropathic pain rats, attributed to the transcriptional repression facilitated by HDAC.^50^ Neuron‐restrictive silencer factor (NRSF) is an epigenetic silencing effector, binding to the neuron‐restrictive silencing elements (NRSE) within the promoter region of genes, such as Nav1.8, Kv4.3 and μ‐opioid receptor genes.^51^ It was suggested that nerve injury upregulates the expression of NRSF in DRG neurons through increased acetylation of histone H4 in the NRSF promoter region, facilitating the binding of NRSF to the NRSE and recruiting ClassI HDAC to deacetylation H3 and H4 histones at NRSE within Nav1.8 gene (SCN10A), resulted in more condensed hetero‐chromatin structure, thereby epigenetic silencing of Nav1.8 gene.^52^ Kv1.2 and Kv4.3 are critical subunits in the Kv channel family in DRG neurons and were downregulated after peripheral nerve injury.^53^, ^54^, ^55^ A study demonstrated that the aberrant expression of Kv1.2 was associated with the upregulation of HDAC2, as supported by the co‐localization of Kv1.2 with HDAC2 in DRG neurons and enhanced binding between histone H3 and Kcna2 (gene encoding Kv1.2) in CCI rats.^24^ More directly, administration of HDAC inhibitor or HDAC2 siRNA effectively restored Kv1.2 levels. Similarly, Uchida et al. found that nerve injury induced silencing of Kv4.3 gene was mediated by increased NRSF binding and histone hypoacetylation at Kv4.3 (KCND3)‐NRSE in DRG neurons.^55^ The voltage‐dependent calcium channel subunit alpha‐2/delta‐1 (α2δ‐1), encoded by Cacna2d1, acts as a glutamate N‐methyl‐D‐aspartate (NMDA) receptors‐interacting protein.^56^ It has been reported that the α2δ‐1 expression in DRG neurons exhibited an early and sustained increase following peripheral nerve injury, enhancing NMDA receptor synaptic trafficking and augmenting neuronal excitability in the spinal dorsal horn.^57^, ^58^ Recently, Zhang et al. indicated that HDAC2 in DRG neurons play a role in the epigenetic modulation of synaptic NMDAR activity in the spinal dorsal horn by regulating α2δ‐1 expression.^59^ They observed a diminished HDAC2 enrichment at the promoter region of Cacna2d1 in DRG of SNL rats, which increased histone acetylation and caused upregulation of α2δ‐1. Moreover, deletion of Hdac2 in DRG neurons induced increased histone acetylation levels at the Cacna2d1 promoter and upregulated α2δ‐1 in DRG, potentiating α2δ‐1‐dependent NMDA receptor activity in the spinal dorsal horn. Surprisingly, Hdac2 knockdown at the spinal cord or Hdac2 conditional knockout in DRG neurons induced long‐lasting mechanical pain hypersensitivity, which challenged the prevailing view of the role of HDACs in facilitating the transition from acute to chronic pain after nerve injury.

Mitochondria are critical organelles for cellular energy production through oxidative phosphorylation. Peripheral sensory neurons, with their long axons, are susceptible to homeostatic mitochondrial functions due to high energy demands.^60^ Mitochondria dysfunction and aberrant mitochondrial transport have been observed in DRG neurons in rats with peripheral neuropathy.^61^, ^62^, ^63^, ^64^ In particular, HDAC6 plays a role in regulating mitochondrial dynamics by deacetylating non‐histone proteins, such as α‐tubulin and heat shock proteins.^65^, ^66^ In a mouse model of cisplatin‐induced neuropathic pain, Krukowski et al. provide evidence that inhibition of HDAC6 reversed cisplatin‐induced mechanical allodynia and reduction in mitochondrial bioenergetics in DRG neurons.^67^ Consistently, Van Helleputte et al. demonstrated that HDAC6 inhibition improved the axonal transport of mitochondria in cultured DRG neurons by increasing α‐tubulin acetylation.^68^

**FIGURE 1:**
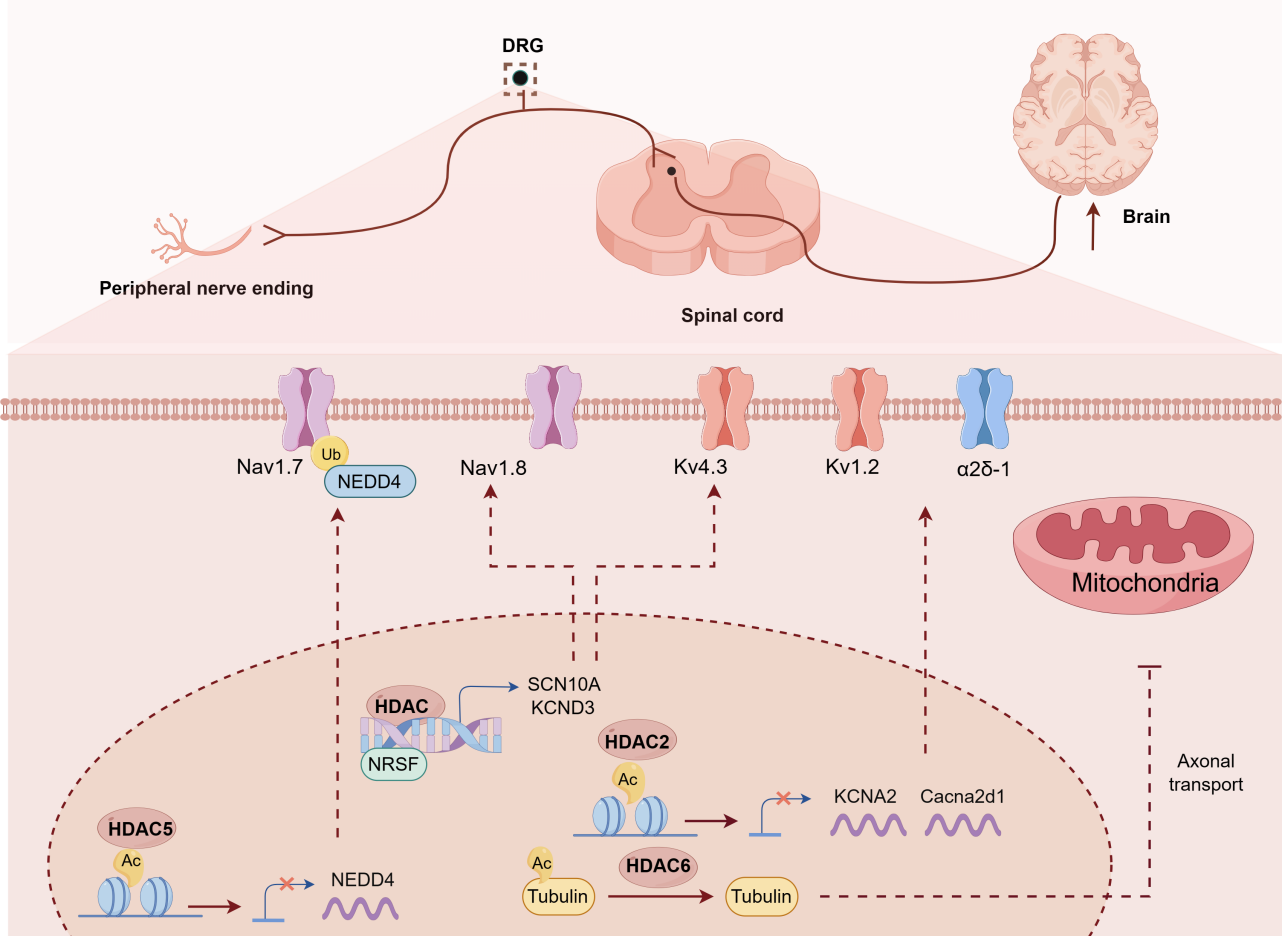
The mechanism of HDACs involved in neuropathic pain at DRG neurons. HDACs binding to the promoter region of pain‐related genes and modulate the acetylation of α‐tubulin, which epigenetically regulating the expression of ion channels and mitochondrial movement in DRG neurons, thereby leading to peripheral sensitization in neuropathic pain.

Collectively, the aforementioned studies indicate that HDACs regulate long‐term pain‐related ion channels gene and mitochondrial function in DRG neurons, leading to peripheral sensitization in neuropathic pain (Figure 1).

### 3.2 Dorsal horn neurons

A large number of studies have suggested that HDACs epigenetically regulate a set of genes associated with neuronal activity in spinal dorsal horn neurons.^22^, ^36^, ^37^, ^41^ Gamma‐aminobutyric acid (GABA) is an inhibitory neurotransmitter synthesized by two isoforms of glutamic acid decarboxylase: GAD65 and GAD67. Downregulation of these enzymes reduces GABAergic inhibition and promotes spinal neuronal hyperexcitability.^69^ Numerous studies have demonstrated that the transcription of GAD65 and GAD67 is epigenetically suppressed through HDACs‐mediated hypo‐acetylation in the Gad1 (gene encoding GAD67) and Gad2 (gene encoding GAD65) promoters.^22^, ^70^, ^71^, ^72^ For example, in a rat model of CCI‐induced neuropathic pain, Quyang et al. found that knockdown of HDAC2 in the spinal cord partially reversed mechanical and thermal hyperalgesia by a mechanism that upregulates GAD65 expression.^22^ Additionally, another study reported that the decreased acetylation in the promoter of Gad1 and Gad2 in the spinal cord of pSNL rats resulted from increased spinal neuronal HDAC5 nuclear translocation.^71^ SRY‐box‐containing gene 10 (SOX10) is a transcription factor that maintains neurons and promotes axon regeneration.^73^ It was recently reported that the secretion of SOX10 in spinal dorsal neurons was positively regulated by HDACs through direct binding to its promoter region, leading to spinal neuronal sensitization.^28^, ^37^ Gu et al. showed that knockdown of spinal HDAC5 and SOX 10 decreased spinal neuronal sensitization markers in pSNL‐induced neuropathic pain.^37^ Conversely, overexpression of spinal HDAC5 induced nociceptive hypersensitivity and increased spinal neuronal sensitization in naïve rats, which was reversed by SOX10 gene knockdown. Similarly, Xie et al. showed that downregulation of HDAC1 attenuated SNL‐induced neuropathic pain by inhibiting SOX 10 expression in spinal dorsal horn neurons.^28^ High‐mobility group box 1 protein (HMGB1), a potent modulator released from sensory neurons, promotes neuronal excitability in the development of neuropathic pain states.^74^ It has been demonstrated that enhanced HMGB1 expression in dorsal horn neurons resulted from the HDAC4 shuttling‐dependent epigenetic modification of the hmgb1 gene in SNL‐induced neuropathic pain rats.^36^ It is well known that metabotropic glutamate receptors (mGluR1) and mGluR5 play a key role in central sensitization and NMDAR regulation.^75^, ^76^ In streptozotocin‐induced diabetic neuropathic pain rats, Zhou et al. demonstrated that SIRT1 mitigated thermal hyperalgesia and mechanical allodynia in DNP rats by reducing mGluR1/5 expression and H3 acetylation level in the Grm1/5 (encoding mGluR1/5) promoter regions.^41^

Oxidative stress and mitochondrial dysfunction in the spinal cord are also key contributors to the induction and maintenance of neuropathic pain through central sensitization.^77^ Transcription factors of the forkhead box class O 3a (FoxO3a) is an important regulator in cellular responses to oxidative stress.^78^ It has been observed that upregulation of spinal SIRT3 increased the FoxO3a level in the spinal cord of DNP‐induced neuropathic pain rats by deacetylating FoxO3a.^42^ Moreover, SIRT3 has been reported to regulate mitochondrial function by acetylation of proteins in mitochondrial, such as cyclophilin D (CypD).^79^ In a very recent study, Yan et al. showed that overexpression of spinal SIRT3 reversed SNI‐induced pain hypersensitivity by deacetylating CypD in the spinal cord.^80^

**FIGURE 2:**
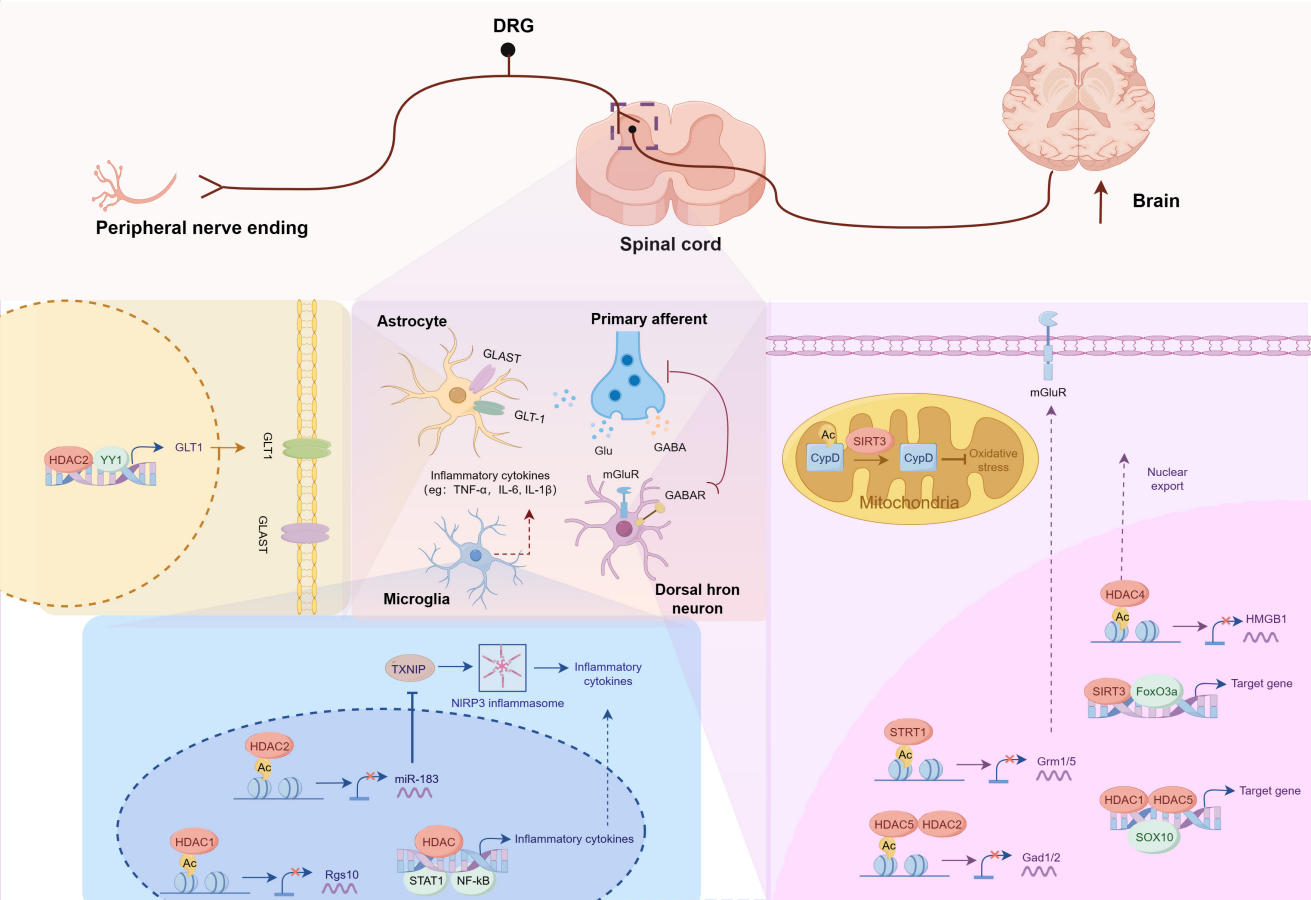
The mechanism of HDACs involved in neuropathic pain at spinal cord. HDACs epigenetically regulates pivotal genes and transcription factors in the spinal cord that modulate neuronal activity in the dorsal horn neuron, pro‐inflammatory cytokines released by microglial, and disturb the regulation of glutamate homeostasis by astrocytes, thus, contributing to the central sensitization during neuropathic pain.

Together with the above results, these studies clearly indicate that HDACs regulate pivotal genes linked to neuronal activity, oxidative stress and mitochondrial function in dorsal horn neurons, contributing to central sensitization during neuropathic pain (Figure 2).

### 3.3 Astrocytes

Astrocytes are the predominant cell type in the CNS and play essential roles in supporting neuronal function and maintaining neurotransmitter homeostasis.^81^ Glutamate is the major excitatory neurotransmitter in the CNS; excess extracellular glutamate leads to neuronal excitotoxicity and, subsequently, neuronal death.^82^, ^83^ Astrocytes primarily achieve the mission of clearing glutamate mediated by glutamate uptake transporters.^84^, ^85^ Evidence has suggested that HDACs might regulates astrocytic glutamate transporter expression in neuropathic pain states.^25^, ^86^ Noteworthy, glutamate–aspartate transporter (GLAST) together with glutamate transporter‐1 (GLT‐1) ensure the absolute majority (80%–90%) of glutamate uptake, and both are predominantly located in spinal astrocytes.^82^ It has been reported the significant downregulation of GLAST and GLT‐1 in the spinal cord leads to the maintenance of pain hypersensitivity in various neuropathic models.^87^, ^88^, ^89^ In a rat model of SNL‐induced neuropathic pain, Hobo et al. showed that treatment with HDAC inhibitor attenuated behavioral hypersensitivity by restoring the downregulated expression of GLAST and GLT‐1 in the spinal cord.^90^ Furthermore, in paclitaxel‐induced neuropathic pain rats, Wang et al. found that the GLT‐1 was significantly decreased in the spinal cord, which was partially reversed by HDAC inhibitor or knockdown of HDAC2.^32^ Notably, Ying‐Yang 1 (YY1), a negative transcriptional regulator of GLT‐1 in astrocytes that undergoes histone deacetylation, has been proposed involved in the modulation of glutamatergic signaling pathway by interacting with HDAC2.^32^, ^91^, ^92^, ^93^ These studies indicate that HDACs disturb the regulation of glutamate homeostasis by astrocytes, eliciting aberrant glutamatergic signaling in the spinal cord during neuropathic pain (Figure 2).

### 3.4 Microglia cells

As the resident macrophages in the CNS, microglia constitute the first line of immune defense, surveilling the surrounding territory in response to injury and emerging as a pivotal mechanism underlying the development of neuropathic pain.^94^ Activated microglia exhibit two distinct polarizations: the proinflammatory phenotype (M1) and anti‐inflammatory phenotype (M2). Following peripheral nerve injury, the microglia undergo a phenotypic transition from M2 to M1 phenotype and subsequently release pro‐inflammatory mediators.^95^ Emerging evidence has illustrated the significant role of HDACs in microglia‐mediated neuroinflammatory in the context of neuropathic pain.^29^, ^31^, ^96^

Valproic acid (VPA), recognized as an HDAC inhibitor, has been proven to inhibit nuclear HDAC3 expression and mitigate SNL‐induced mechanical allodynia by promoting the polarization of microglia toward the M2 phenotype in the spinal cord.^30^ Likewise, Chen et al. also demonstrated that VPA treatment reduced SCI‐induced inflammatory response by promoting the phenotypic shift of microglia from M1 to M2 phenotype and inhibiting microglial activation.^97^ Moreover, Borgonetti et al. demonstrated that administration of HDAC inhibitor suberoylanilide hydroxamic acid (SAHA) inhibited the increased spinal HDAC1 expression and attenuated pain hypersensitivity of SNL mice via suppressing microglia activation in the spinal cord.^29^ Notably, all these studies showed that treatment with HDAC inhibitors inhibited nuclear factor‐kappa B (NF‐kB) p65 nuclear translocation and upregulated expression of the signal transducers and activators of transcription 1 (STAT1), both of which play a critical role in the neuroinflammatory responses triggered by activated microglia.^98^ Additionally, an increased acetylation level of STAT1 and NF‐kB was also observed in the spinal cord following VPA treatment.^30^, ^97^ However, there is currently insufficient conclusive evidence establishing the connection between specific HDAC subtypes, such as HDAC3 and HDAC1, and the genes encoding STAT1 and NF‐kB. Recently, Miao et al. showed that HDAC2 was enriched in the promoter region of miR‐183, which could repress inflammatory responses in the spinal cord of CCI rats via regulating the thioredoxin interacting protein (TXNIP)/NLR family pyrin domain‐containing 3 (NLRP3) inflammasome axis.^23^ Silencing HDAC2 was demonstrated to elevate the acetylation of histone H4 and upregulated the miR‐183 expression in microglial cells.^23^ Similarly, the regulator of G protein signaling (RGS10) functions as a negative regulator of proinflammation cytokines production in microglia.^31^, ^99^ Alainyah et al. found that activated microglia‐induced transcriptional silencing of Rgs10 expression and proinflammatory cytokines production, which was reversed by HDAC inhibitor treatment. Furthermore, they demonstrated that the suppressed transcription of Rgs10 in activated microglia was associated with increased HDAC1 recruitment at the Rgs10 promoter.^31^

In summary, these findings suggest that HDACs regulate genes associated with microglia activation and polarization in the spinal cord, promoting neuroinflammation during neuropathic pain (Figure 2).

### 3.5 Macrophages and T cells

Ample evidence has proved the engagement of macrophage and T cells in the maintenance of neuropathic pain.^100^, ^101^, ^102^ Conventionally, macrophages are classified as M1‐like macrophages and M2‐like macrophages. CD206, also known as macrophage mannose receptor 1 (MRC1), is a marker of M2‐like macrophages, which produce anti‐inflammatory cytokines such as interleukin‐10 (IL‐10) and specialized pro‐resolving mediator necessary for pain relief.^101^, ^102^ A recent study showed the involvement of HDA6 in the regulation of MRCI‐positive macrophages during neuropathic pain.^103^ It has been observed that treatment with HDAC6 inhibitor reversed cisplatin‐induced neuropathic pain and suppressed IL‐10 mRNA expression in the spinal cord, which was blocked by the depletion of spinal CD206‐positive macrophages without affecting microglia. Similar to macrophages, T cells encompass a range of diverse subsets and infiltrate in damaged peripheral nerves, DRG, and the spinal cord after nerve injury. Among the multiple subsets of T cells, regulatory T cells (Trges) exert pain‐suppressing functions by suppressing proinflammation immune response.^104^, ^105^ Evidence provided proof that SIRT1 promoted the differentiation of CD4^+^ T cells toward Treg cells and limited inflammation associated with neuropathic pain.^106^ These findings suggest that HDACs orchestra immune response modulated by macrophage and T cells in the development of neuropathic pain. However, research in this area is limited, making it a promising direction for proactive exploration in the future.

## 4 THE HDAC INHIBITORS AS POTENTIALLY THERAPEUTIC ANALGESICS

In light of the pivotal role of HDACs in the pain process, targeting HDACs emerges as a promising therapeutic strategy. A great deal of research has proved that pharmacological modulation of histone acetylation by HDAC inhibitor (HDACi) reversed the nociceptive response in various neuropathic pain models. To date, these compounds exhibit varying levels of selectivity for distinct HDAC classes, with the majority of targeting Class I HDACs and being chemically zinc‐dependent. Here, we summarize the therapeutic effects of various pan‐HDAC inhibitors in rodent models of neuropathic pain (Table 2).

**TABLE 2:** Therapeutic potential of HDAC inhibitors in neuropathic pain.

Inhibitors	Targets	Model	Treatment strategy	Effect	References
SAHA	Class I and II	SNL Rats	Intrathecal injection (100 nmol/day or 200 nmol/day) for 12 days, starting 5 days before SNL	PWT ⬆	107
		SNI Mice	Intrathecal or intranasal administered on post‐surgical day 7 (3 or 10 μg)	PWT, PWL ⬆	29
		SCI Mice	Intrathecal injection (3 or 10 μg) 15 min before behavior tests	PWT, PWL ⬆	26
VPA	Class I and II	SNL Rats	Intrathecal injection (300 mg/kg/day) for 3 days after SNL	PWT ⬆	30
		SNL Rats	Orally administered twice a day (300 mg/5 mL/kg) from day 0 to day 21	PWT ⬆	109
		CIPN Rats	Intraperitoneal injection (200 mg/kg) at day 7 to14 after CIPN	PWT ⬆	32
		DNP Mice	Orally administered (25 or 50 mg/kg) for 5 weeks per day	PWT, TWL ⬆	110
Sodium butyrate	Class I and II	CCI Rats	Orally administered (200 and 400 mg/kg) starting from day 1 to day 14	PWT, TWL ⬆	112
		CCI Mice	Orally administered (200 mg/kg) at day 4 to 14 after CCI	PWT, TWL ⬆	113
TSA	Class I and II	CCI Rats	Intrathecal injection (2 or 10 μg) for 3 consecutive days after CCI	PWT, TWL ⬆	22
MS‐275	Class I	SNL Rats	Intrathecally infusion (30 or 60 nmol/day) for 7 consecutive days after SNL	PWT ⬆	116
ACY‐1215	HDAC6	SNL Rats	Intraperitoneally injected (25 mg/kg/day) after SNL for 21 consecutive days	PWT ⬆	118
		CIPA Mice	Orally administration (30 mg/kg) for 2 weeks, starting at 3 days after the last dose of cisplatin	PWT ⬆	67
ACY‐1083	HDAC6	CIPA Mice	Intraperitoneal injection (10 mg/kg) for 7 days, starting at 3 days after the last dose of cisplatin	PWT ⬆	67, 103
ACY‐738	HDAC6	SNI Mice	Intraperitoneal injection (5 mg/kg) for 40 days after SNL	PWT ⬆	120
LG325	HDAC1	SNI Mice	Intrathecal injection (1 and 5 μg) on day 7 post‐SNI	PWT ⬆	122
ZOE	HDAC1	SNI Mice	Oral administration (200 mg/kg/day) for 7 days starting from 3 days after SNI	PWT, TWL ⬆	124
ZNG	HDAC1	SNI Mice	Orally administration (10 mg/kg) on the 10th day after SNI	PWT, TWL ⬆	125

Abbreviations: CCI, chronic constriction injury; CIPA, chemotherapy‐induced peripheral neuropathy; DNP, diabetic neuropathic pain; PWL, paw withdrawal latency; PWT, paw withdrawal threshold; SAHA, suberoylanilide hydroxamic acid; SCI, spinal cord injury; SNI, spared nerve injury; SNL, spinal nerve ligation; TSA, trichostatin A; TWL, thermal withdrawal latency; VPA, valproate acid; ZNG, zingiberene; ZOE, zingiber officinale Roscoe rhizome extract.

### 4.1 Zinc‐dependent inhibitors

#### 4.1.1 SAHA

SAHA, a type of HDACi that inhibits both Class I and Class II HDACs, was proved for the treatment of cancer. An increasing body of evidence has verified the analgesia effects of SAHA in neuropathic pain states.^26^, ^50^, ^107^, ^108^ For example, Feng et al. found that daily intrathecal injection (i.t.) of SAHA (100 nmol/day or 200 nmol/day) reversed the mechanical allodynia in SNL‐induced neuropathic pain rats.^107^ Likewise, Borgonetti's group demonstrated intranasal administration of SAHA (3 and 10 μg) dose‐dependently ameliorated mechanical and thermal pain hypersensitivity in SNI mice.^29^ Furthermore, in SCI‐induced central neuropathic pain, Wang et al. showed that intraperitoneally administration (i.p.) with SAHA (1–10 μg) ameliorated mechanical and thermal hyperalgesia in SCI mice.^26^

#### 4.1.2 VPA

VPA, or valproic acid, and valproate sodium are licensed for treating epilepsy and bipolar disorder. Several laboratories have investigated its efficacy on neuropathic pain and indicated that VPA has promising anti‐inflammatory and analgesic properties by regulating the gene transcription through the inhibition of HDACs.^90^, ^109^ In a rat model of SNL‐induced neuropathic pain, Hobo et al. showed that repeated oral administration of VPA (300 mg/kg, twice a day) significantly increased the mechanical withdrawal threshold of SNL rats.^90^ Similarly, another group showed that VPA (300 mg/kg, i.p.) treatment efficiently attenuated SNL‐induced mechanical allodynia.^30^ Recently, Elsherbiny et.al showed that repeated oral administrated of valproate sodium (25 and 50 mg/kg) ameliorated tactile allodynia and thermal hyperalgesia in a mouse model of alloxan induced‐diabetic neuropathic pain.^110^

#### 4.1.3 Sodium butyrate

Sodium butyrate is an HDAC inhibitor selectively targeting subtypes I and IIa.^111^ Kukkar et al. revealed that oral administration of sodium butyrate at doses of 200 and 400 mg/kg for 14 days attenuated CCI‐induced pain‐like behaviors.^112^ Similarly, Russo et al demonstrated that repeated oral administrations of butyrate (200 mg/kg) reduced mechanical and thermal hyperalgesia in CCI mice.^113^

#### 4.1.4 TSA

Trichostatin A (TSA) is originally an antibiotic and was further discovered by Yoshida et al. to function as a pan‐HDAC inhibitor.^11^ Ouyang et al. have revealed the ameliorative effect of TSA in a dose‐dependent manner in CCI rats.^22^ They showed that administration of TSA (2 or 10 μg, i.p.) after CCI surgery partially reversed the decreased mechanical and thermal threshold in CCI rats. Moreover, TSA was found to increase brain‐derived neurotropic factor (BDNF) expression in the sciatic nerves of diabetic mice and improve nerve conduction function.^114^ Considering its high toxicity, TSA is not widely used for pain management, but is utilized as a template drug for developing novel HDAC inhibitors.

#### 4.1.5 MS‐275

MS‐275 is a potent selective inhibitor of class I HDACs and exhibits fewer side effects and toxicity.^115^ In a rat model of pSNL‐induced neuropathic pain, Denk et al. demonstrated that continuous intrathecally infusion with MS275 (30 or 60 nmol/day) via an osmotic pump significantly attenuated mechanical and thermal hypersensitivity by targeting HDAC1.^116^

### 4.2 Isoform‐specific HDAC inhibitors

#### 4.2.1 HDAC6 specific inhibitors

ACY‐125 is an orally bioavailable HDAC6 inhibitor, currently used in clinical for treating multiple myeloma and lymphoma, exhibiting over10‐fold selectively against HDAC6 than Class I HDACs.^117^ In a mouse model of cisplatin‐induced neuropathic pain, oral administration of ACY1215 (30 mg/kg) for 2 weeks effectively reversed cisplatin‐induced mechanical allodynia.^67^ Recently, Chen et al. showed that continuous treatment with ACY‐1215 (25 mg/kg/d, i.p.) after operation attenuated mechanical allodynia, cognitive impairment and depressive‐like behavior in SNL‐induced neuropathic pain rats.^118^ ACY‐1083, a highly selective HDAC6 inhibitor, exhibits 260‐fold selectivity toward HDAC6 over other classes. Krukowski's lab has demonstrated the prominent analgesic effect of ACY‐1083 in chemotherapy‐induced neuropathic pain.^67^, ^103^ They revealed that posttreatment with ACY‐1083 (10 mg/kg, i.p.) relieved established cisplatin‐induced mechanical allodynia, spontaneous pain and numbness. Moreover, pretreatment with ACY‐1083 also prevented the development of mechanical hyperalgesia. ACY‐738 is a newly developed small molecule inhibitor of HDAC6 that can penetrate the brain, with strong potency and a selectivity of 60 to 1500‐fold over class I HDACs.^119^ Sakloth et al. demonstrated the effective antiallodynic effects of ACY‐738 both in male and female mouse models of SNI‐induced neuropathic pain.^120^

#### 4.2.2 HDAC1‐specific inhibitors

LG325 displays selective inhibitory activity against HDAC1. Sanna et al. found that a single dose of LG325 (5 μg, i.t.) treatment presented a prolonged anti‐hyperalgesia efficacy in SNI mice, comparable to the effects of pregabalin and morphine.^121^, ^122^ Notably, the anti‐nociceptive effect of LG325 was attained without any visible side effects or impairment of locomotor activity, even at the highest effective dose.

### 4.3 Natural products

Zingiber officinale Roscoe (Zingiberaceae), also referred to as ginger, is widely used as a spice. Zingiber officinale rhizome has been used to treat inflammatory disease.^123^ Recently, the standardized Zingiber officinale Roscoe rhizome extract (ZOE) and Zingiberene (ZNG) were identified as new non‐zinc‐dependent inhibitors of HDAC Class I, both of which reduce neuroinflammation through HDAC1 inhibition and have therapeutic application in neuropathic pain. In a mouse model of SNI‐induced neuropathic pain, Borgonetti et al demonstrated that oral administration of ZOE (200 mg/kg) and ZNG (20 mg/kg) significantly reduced thermal hyperalgesia and mechanical allodynia in SNI mice by reducing the expression of HDAC1 in the spinal cord.^124^, ^125^ Specifically, the non‐zinc‐dependent inhibitors have higher selectivity and better safety profile than other HDAC inhibitors.

## 5 CONCLUDING REMARKS AND PERSPECTIVE

In this comprehensive review, we delved into the cellular and molecular mechanism of HDACs underlying the pathophysiology of neuropathic pain. These studies revealed that abnormally elevated HDACs in DRG and spinal cord promote neuronal excitability and neuroinflammation by epigenetically modulating pivotal gene expression in neuronal and immune cells, thus contributing to peripheral and central sensitization in the progression of neuropathic pain. However, the majority of research are descriptive and correlational. Alterations in HDAC levels across the entire tissue may not accurately reflect the abundance of HDACs locally at the pro‐ and anti‐nociceptive genes during pain progression. With a better and holistic understanding of the HDAC underpinning neuropathic pain, it is urgent to identify the occupancy of binding sites of HDACs on individual gene promoters to accurately elucidate the role of specific HDAC subtypes in governing gene transcription associated with neuropathic pain.^6^ Additionally, our understanding of the epigenetic process in the brain regions and circuits associated with pain perception remains comparatively restricted. Future endeavors should prioritize bridging this knowledge gap concerning the expression pattern of HDACs and their target gene within the brain area. Importantly, epigenetic regulators are shared across multiple cell types and tissues, research focus on single‐cell epigenetics will be essential to advance our comprehension in the pathogenesis of neuropathic pain.^126^

In contrast to the established understanding of HDAC promoteing neuropathic pain, there are few studies indicating that histone acetylation actually induces pain. For example, in the CCI rat model of neuropathic pain, inhibition of HAT activity reduced pain hypersensitivity and decreased cyclooxygenase‐2 (COX2) expression in the spinal cord.^127^ Moreover, it has been demonstrated that HAT inhibitors attenuated pSNL‐induced thermal hyperalgesia by suppressing acetylation of histone H3 at the promoters of inflammatory mediators, such as CXC chemokine ligand type 2 (MIP‐2) and CXC chemokine receptor type 2 (CXCR2), in the neutrophils and macrophages of the injured nerves.^128^ It is important to recognize that the neuropathic pain conditions typically manifest progressively over time, and the interplay between histone acetylation and deacetylation during the progression of neuropathic pain is dynamic and intricate. The contradictory findings observed in experimental studies may be attributed to the differential regulation of transcriptional processes by HDAC and HAT across distinct temporal and spatial dimensions. However, limited attention has been paid to the timescale of these changes, which emphasizing the importance of considering the temporal dynamics of epigenetic process underlying neuropathic pain pathogenesis.

Furthermore, we have extensively discussed various therapeutic drugs aimed at Whereas, many of these drugs approved for clinical lack isoform selectivity, which raises concerns about their toxicity and off‐target effects. Thus, it is of great interest to design isoform‐specific HDACi might maximize drug efficacy and safety, while minimize the potential side effects. More importantly, strategies to inhibit HDACs also should target different cell types independently.^19^, ^129^ Nevertheless, the analgesic effect of HDACi in neuropathic pain is primarily based on rodent models and is still in the preclinical stage. There is an impendency for clinical trials to establish the efficacy of HDAC inhibitors on patients suffering from neuropathic pain.

## AUTHOR CONTRIBUTIONS

Original draft preparation, ZWC; review and editing, ZXW; visualization, JB and YSC; supervision, ZCX and ZKW; funding acquisition, LBW. All authors read and approved the final manuscript.

## FUNDING INFORMATION

This work was supported by the National Natural Science Foundation of China (Nos.2101103482).

## CONFLICT OF INTEREST STATEMENT

The authors declare no competing interests.

## Data Availability

Data sharing is not applicable to this article as no new data were created or analyzed in this study.
